# Serial repetitive nerve stimulation studies in organophosphorus poisoning indicate two distinct pathophysiological processes occur at the neuromuscular junction in the intermediate syndrome

**DOI:** 10.1080/15563650.2024.2343744

**Published:** 2024-05-13

**Authors:** Nicholas A. Buckley, Pradeepa Jayawardane, Vajira Weerasinghe, Andrew H. Dawson, Tharaka Lagath Dassanayake

**Affiliations:** a South Asian Clinical Toxicology Research Collaboration (SACTRC), Faculty of Medicine, University of Peradeniya, Peradeniya, Sri Lanka; b Translational Australian Clinical Toxicology (TACT) Research Group, Faculty of Medicine and Health, The University of Sydney, Sydney, NSW, Australia; c Department of Pharmacology, Faculty of Medical Sciences, University of Sri Jayewardenepura, Nugegoda, Sri Lanka; d Department of Physiology, Faculty of Medicine, University of Peradeniya, Peradeniya, Sri Lanka

**Keywords:** Intermediate syndrome, organophosphorus poisoning, nerve conduction studies, repetitive nerve stimulation, toxic mechanisms

## Abstract

**Introduction:**

Intermediate syndrome is an important cause of respiratory failure following acute organophosphorus pesticide poisoning. The objective of this study was to examine the pathophysiology of this syndrome by analysis of sequential repetitive nerve stimulation studies in patients with acute organophosphorus pesticide poisoning.

**Methods:**

Thirty-four consenting symptomatic patients with acute organophosphorus pesticide poisoning with intermediate syndrome (*n* = 10) or a milder *forme fruste* intermediate syndrome (*n* = 24) were assessed prospectively with daily physical examination and repetitive nerve stimulation done on the right and left median and ulnar nerves. The compound muscle action potential at 1, 3, 10, 15, 20 and 30 Hertz was measured with a train of ten stimuli. The amplitudes of the resulting stimuli were normalized to the first stimulus (100 per cent) and plotted against time. The decrease in the area under the curve of all the second stimulus compound muscle action potentials in the first 0.3 seconds was measured as a means of quantifying the refractory block. The decrease in the area under the curve under the 10, 15, 20 and 30 Hertz compound muscle action potentials relative to this pooled second stimulus compound muscle action potentials-area under the curve indicated the extent of additional rate-dependent block (decreasing compound muscle action potential-area under the curve over the first 0.3 seconds after the first stimulus with increasing Hertz).

**Results:**

These new measurements strongly correlated with the severity of weakness. Refractory block was seen in most patients but was more severe in those with intermediate syndrome than those with *forme fruste* (partial) intermediate syndrome (median 55 per cent versus 16 per cent, *P* = 0.0001). Similar large differences were found for rate-dependent block (30 per cent versus 7 per cent, *P* = 0.001), which was uncommon in *forme fruste* intermediate syndrome but found in nine out of 10 patients with intermediate syndrome. Rate dependent block was generally only observed after 24 hours. The simplest strong predictor was total block at 30 Hertz repetitive nerve stimulation (89 per cent [interquartile range 73 to 94 per cent] versus 21 per cent [4 to 55 per cent]; *P* < 0.0001), which was very similar to total block calculated by summing other calculations.

**Discussion:**

These findings likely represent depolarization and desensitization block from prolonged excessive cholinergic stimulation but it is not clear if these are from pre- or post-synaptic pathology. An animal model of intermediate syndrome with repetitive nerve stimulation studies might enable a better pathophysiological understanding of the two types of block.

**Limitations:**

The limited number of repetitive nerve stimulation studies performed were sufficient to demonstrate proof-of-concept, but further studies with more patients are needed to better define the correlates, clinical relevance and possible diagnostic/prognostic roles for the use of this technique.

**Conclusion:**

There are two easily distinguishable pathophysiological abnormalities in the neuromuscular block in intermediate syndrome. While they often coincide, both may be observed in isolation. The total and rate-dependent block at 30 Hertz are strongly associated with more severe weakness.

## Introduction

Self-poisoning using pesticides is a major but under-recognized global health problem. Each year, it is estimated that 110,000-168,000 people die from deliberate ingestion of pesticides. These deaths are responsible for about a fifth of the global burden of illness from suicide [1]. These estimates have halved from those from twenty years ago [2,3], reflecting the success of widespread bans of highly hazardous pesticides in China and other parts of Asia [1]. Organophosphorus insecticides are the most important class of pesticides in this global public health problem [2]. Organophosphorus poisoning clinical syndromes are often characterized based on their onset:

1. the acute cholinergic syndrome;
2. the intermediate syndrome, a syndrome of muscular paralysis which develops after 1-4 days and may last for some weeks;
3. the organophosphorus-induced delayed polyneuropathy, which is much less common and usually comes on after some weeks and may be permanent.

The term intermediate syndrome highlighted the important observation that apparently well patients who had recovered from acute cholinergic effects could rapidly develop severe weakness [4]. The cholinergic symptoms frequently overlap such that a clearly definable intermediate syndrome may not be apparent, and the term is probably more widely used to describe any severe muscle weakness that has an onset around 1 to 4 days after organophosphorus poisoning. Intermediate syndrome in acute organophosphorus poisoning carries a high risk of death because of its associated delayed onset respiratory failure [5,6].

The intermediate syndrome was defined as a syndrome of muscular weakness affecting predominantly the proximal limb muscles and those supplied by the cranial nerves [4]. The pathophysiology of the intermediate syndrome is not clearly understood [6–9] but is generally believed to result from a persistent excess of acetylcholine at the neuromuscular junction [7,8]. Neuromuscular transmission has been recorded in patients with intermediate syndrome using repetitive nerve stimulation and single fiber electromyography [7,10–12]. Repetitive nerve stimulation most commonly shows a decrement-increment response that is seen in few other conditions [7,11,12].

We previously demonstrated a number of new findings in our studies on intermediate syndrome. We prospectively assessed the neuromuscular junction with repetitive nerve stimulation in patients who were at risk of developing intermediate syndrome to gather more information on the pathophysiology of intermediate syndrome and to look for any predictors of the syndrome that would be relevant clinically and in research. We demonstrated firstly that intermediate syndrome could occur before 24 h and even after 96 h [5,12]. Secondly, we demonstrated that the neuromuscular junction effects were better conceptualized as a continuous spectrum of severity through which patients progressed and then resolved over some days. We also noted that there was sometimes a marked change in the repetitive nerve stimulation morphology from a decrement-increment pattern to a progressive decrement at the time when weakness was so severe that respiratory function became compromised [12].

A previous study with the anticholinesterase neostigmine in rodents with micro-electrode monitoring of membrane potentials suggest that low doses predominantly cause post-synaptic desensitization that tends to counteract depolarizing block in turn leading to increment pattern following the initial decrement (i.e., a decrement-increment response) [13]. At greater concentrations of anticholinesterases, desensitization seems to intensify the blockade leading to a decrement response in repetitive nerve stimulation [13]. As such, there is no strong reason to believe that pre- and post-synaptic pathophysiology is mutually exclusive. Further, this model observed these effects acutely, and it is unclear whether this represents the same pathophysiology as the delayed onset intermediate syndrome.

These two patterns and the same correlation with the extent of red blood cell acetylcholinesterase activity have been found in repetitive nerve stimulation in humans with organophosphorus poisoning [14]. The most common finding in intermediate syndrome is a decrement-increment on repetitive nerve stimulation that is most obvious at higher frequencies [12,14]. The decrement response is associated with more severe red blood cell acetylcholinesterase inhibition and intermediate syndrome features, but the overlap and transition between these two phenotypes is not clear. The lowest compound muscle action potential in the train with decrement increment responses is nearly always the second. There would be advantage in methods whereby a pure decrement-increment response due to a prolonged initial depolarization could be separated from potentially concealed decrement responses indicating desensitization. Previously, better methods to indicate the extent of the decrement block have been suggested, such as the ratio of the ninth to the first compound muscle action potential at high repetitive nerve stimulation frequencies [14]. That potentially improves risk assessment but still combines the two types of block in one measure [14]. We aimed to develop a technique that might be used to measure the onset and associations of the second decrement type of neuromuscular junction block more accurately. We herein describe a method of separating and quantifying rate-independent (refractory) block and rate-dependent neuromuscular junction block (increasing block with increasing rates of stimulation).

We then aimed to examine whether these two distinct pathophysiological abnormalities were detectable on repetitive nerve stimulation in both intermediate syndrome and the *forme fruste* intermediate syndrome (patients with some intermediate syndrome features but not the full classical syndrome) and whether either block was more strongly associated with severe weakness and subsequent high risk of respiratory failure.

## Methods

Patients in this study are a subset from a prospective observational study of symptomatic patients poisoned with organophosphorus insecticides and recruited from Nuwara Eliya General Hospital, Nuwara Eliya, Sri Lanka, from May 2005 to April 2006 and from Teaching Hospital, Peradeniya, Sri Lanka, from May 2006 to December 2006 [12]. The study had the approval of the Human Research Ethics Committees of the University of Peradeniya, Sri Lanka and the Australian National University. Informed written consent was obtained from all the study patients.

The inclusion criteria were admission within 24 h of ingestion of an organophosphorus insecticide and signs of systemic intoxication. Patients less than 15 years of age and pregnant patients were excluded. Organophosphorus poisoning was confirmed by the history from the patient and/or relatives, containers brought to hospital, records in patient-transfer forms, characteristic smell in the breath and clinical features typical of organophosphorus poisoning. Additional biochemical evidence of organophosphorus poisoning (serum organophosphorus concentration or red blood cell acetylcholinesterase activity) was available in some of the original patients [12]. Our definition for intermediate syndrome was significant muscle weakness (<4/5 Medical Research Council [MRC] grading) in at least three of the following muscle groups (extraocular, neck flexor, proximal limb, and facial) observed at least 24 h after ingestion of an organophosphorus insecticide [4,12]. *Forme fruste* intermediate syndrome patients had weakness of lesser extent or severity but in the same time frame.

As per institutional practices at the time, patients were treated with a 10-15 mg bolus dose of atropine followed by 10-15 mg atropine infusion in 0.9% normal saline over about 12 h, with the infusion rate adjusted according to clinical response. Pralidoxime 1 g six hourly was administered for 48 h as a slow intravenous injection. All patients in this study received atropine, and most also received pralidoxime.

Electrophysiological tests were done at the bedside using a portable Medelec Synergy electromyography (EMG) machine (software version 11). Repetitive nerve stimulation was performed on the right and left median and ulnar nerves, with recording electrodes placed over the abductor policis brevis and abductor digiti minimi muscles, respectively. Nerves were stimulated superficially with supramaximal stimuli by a stimulator placed over the respective nerves at the wrist. Recordings were done with TECA NCS disposable bar electrodes using the belly tendon configuration. The “stimulation” hand was immobilized manually to prevent movement artefacts. A 50 Hz notch filter was used. Repetitive nerve stimulation studies were done with a train of 10 supramaximal stimuli of 0.1 ms duration at 1, 3, 10, 15, 20 and 30 Hz frequencies. There was at least a 15 s interval between two trains of stimuli.

## Results

We examined repetitive nerve stimulation in 10 patients with intermediate syndrome. Late onset (>24 h) respiratory failure requiring supported ventilation developed in five of these intermediate syndrome patients. We compared them against 24 patients with the *forme fruste* intermediate syndrome. These patients developed varying degrees of weakness involving the neck flexors, proximal limb muscles and muscles supplied by the motor cranial nerves and had some characteristic electrophysiological patterns at some time point but did not meet the standard criteria of severe weakness in at least three muscle groups to qualify for a diagnosis of classical intermediate syndrome [12].

Table 1 shows presenting disease severity, admission characteristics and the treatment details of patients with intermediate syndrome, and those with *forme fruste* intermediate syndrome.

**Table 1.** Presenting disease severity, admission characteristics and the treatment details of the ten patients with intermediate syndrome and 24 patients with *forme fruste* intermediate syndrome.

	Intermediate syndrome (*n* = 10)	*Forme fruste* intermediate syndrome (*n* = 24)
Median age (IQR) in years	27 (22 to 34)	27 (19 to 36)
Male gender, *n* (%)	7 (70%)	22 (92%)
Median time (min) to admission (IQR)	155 (77 to 435)	63 (30 to 235)
Median length of stay in days (IQR)	11 (8 to 25)	5.5 (5 to 8)
Intubated, *n* (%)	5 (50%)	3 (12.5%)
Median duration of intubation in h (IQR)	208 (147–570)	139 (2–143)
Median minimum red blood cell acetylcholinesterase activity mU/µmol Hb (IQR) (intermediate syndrome *n* = 6, *forme fruste* intermediate syndrome *n* = 21)	17 (5.6 to 32.4)	27 (15 to 57)
Median chlorpyrifos concentrations μM (intermediate syndrome *n* = 5, *forme fruste* intermediate syndrome *n* = 21) (IQR)	2.06 (1.03 to 5.1)	3.28 (1.62 to 4.45)

From the original patients who had either intermediate syndrome (*n* = 10) or *forme fruste* intermediate syndrome (*n* = 33), we selected patients with repetitive nerve stimulation studies appropriate for further analysis; 159 repetitive nerve stimulations were measured on these 43 patients. To be included in this new analysis, repetitive nerve stimulation at greater than 10-15 Hz were required and only 24 of 33 patients with *forme fruste* intermediate syndrome had these measurements. There were 127 repetitive nerve stimulations (from 34 patients) with a full or near full complement of frequencies from 1 to 30 Hz. These were used to show the changes in the block over time. Of the 127 repetitive nerve stimulations, 87 showed at least a minimal decrement (second stimulus compound muscle action potential at 30 Hz <95% of first stimulus compound muscle action potential) and 55 repetitive nerve stimulation studies from 27 patients showed a larger block (second stimulus compound muscle action potential at 30 Hz <70% of first stimulus compound muscle action potential). In these, it was relatively simple to separate out if there were different components. The process of developing this method was based on an analysis of these 55 readings (but then later applied to all 127 repetitive nerve stimulation studies from the 33 patients).

**Figure 1.**
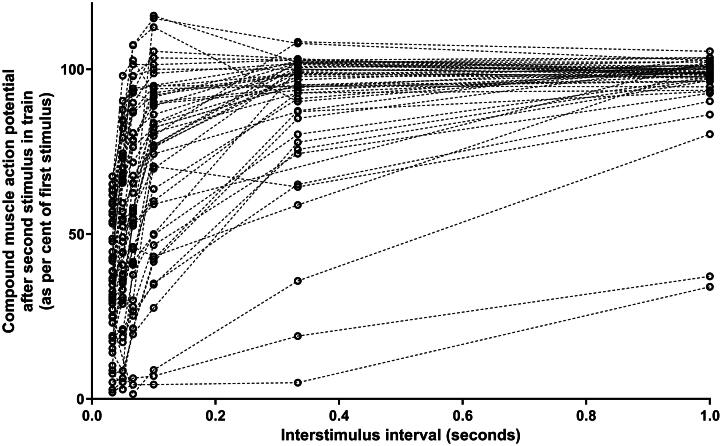
Refractory block from 55 repetitive nerve stimulation readings in 27 patients. These are all the repetitive nerve stimulation studies where the compound muscle action potential after the second stimulus was <70% of the first stimulus. The extent of block with longer intervals to the second stimulus is also shown to indicate the rate of recovery.

In Figure 1, we show these 55 readings with a plot of the amplitude of the second stimulus in each train of ten against the inter-stimulus interval (time from the first stimulus). Given the Hz are different it follows the second stimuli are at different times after the first stimulus (i.e., 0.033, 0.05, 0.067, 0.1, 0.33 and 1.0 s after the first stimulus). It can be seen that the pattern is an obvious refractory block after a single stimulus that appears to recover with a simple one phase association. A mathematical relationship can be modelled on the assumption that the rate of return to the normal polarized state will be proportional to the degree of depolarization – i.e., there will be an exponential decline in the extent of depolarization with time [this equation would be second stimulus_t_ = second stimulus_0.033 s_ + (100% − second stimulus_0.033 s_) * (1−e^(-kt)^)]. This is also consistent with the profile of recovery shown in other less severe refractory blocks in more invasive electrophysiological studies [15].

**Figure 2.**
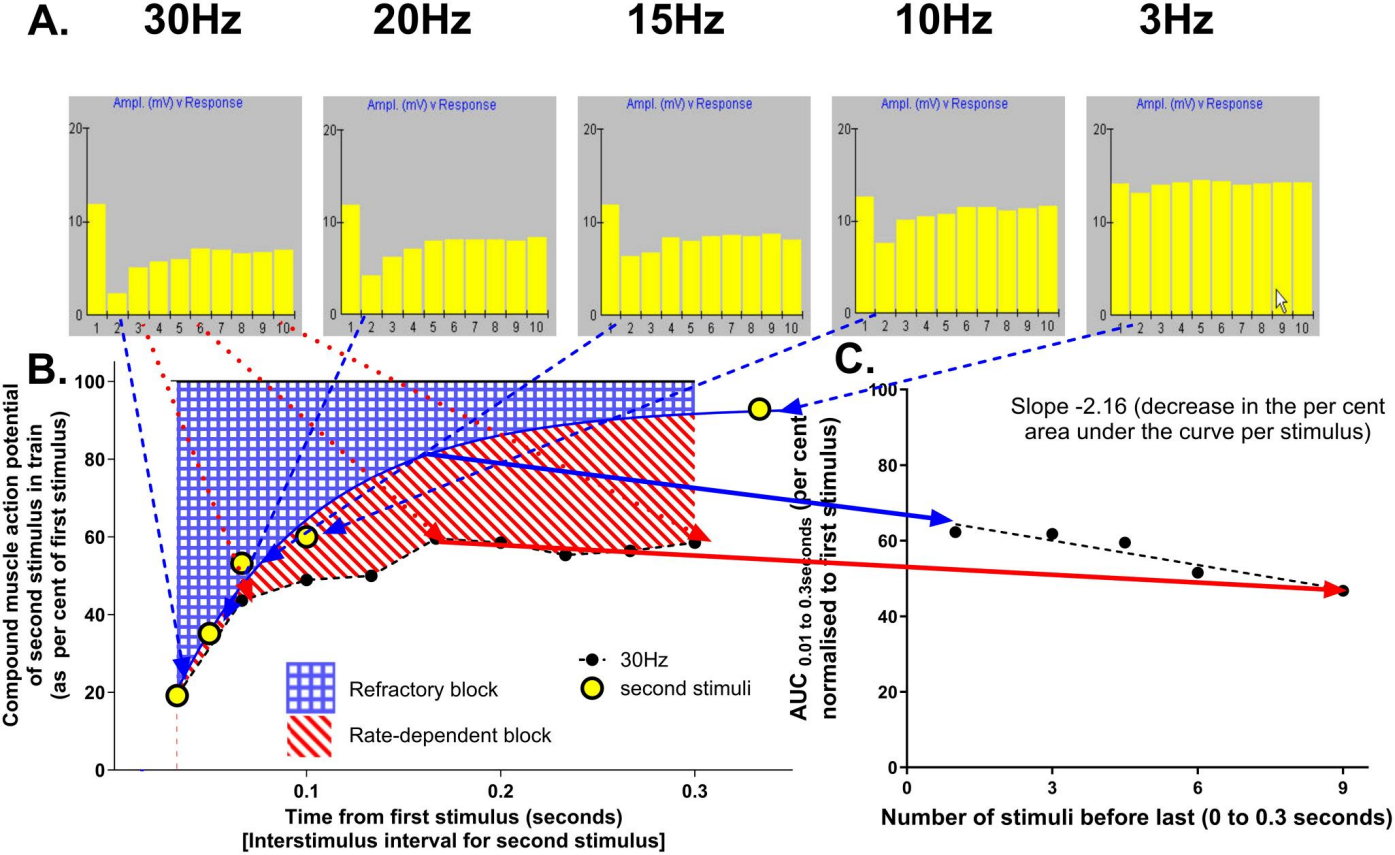
Method of analysis of repetitive nerve studies to differentiate refractory and rate-dependent contributions to neuromuscular block in the intermediate syndrome. (A) The original repetitive nerve stimulation studies at 3,10,15,20 and 30 Hz. (B) The calculation of area under the curve for pooled second stimuli indicating refractory block, and the loss of area under the curve at 30 Hz indicating the further rate-dependent block. (C) Further quantification of the rate-dependent decline in area under the curve by linear regression.

From this observation, Figure 2 demonstrates the analysis that was then applied to all the 127 repetitive nerve stimulation studies with a sufficient range of frequencies. The circled second stimulus in the chain of ten of all the repetitive nerve stimulations between 1 and 30 Hz indicates the time course and severity of the refractory block after the first stimulus. The recovery from this block can be further quantified by fitting the solid line (a non-linear fit of the one phase association of all the second stimuli – second stimuli_t_ = second stimuli_0.033 s_ + (100% − second stimuli_0.033 s_) * (1−e^(-kt)^)). The area above this curve (blue hatched shading) was used as a means of quantifying the reduction in compound muscle action potentials due to refractory block (a 'normal’ individual’s reading would be roughly horizontal at 100%). The 30 Hz readings are also shown in Figure 2 to indicate the presence of a further rate-dependent decrease in area under the curve (i.e., red diagonal striped area) relative to the simple refractory block. For clarity, only this line is shown, but the area indicating rate-dependent block was also calculated for 10, 15 and 20 Hz repetitive nerve stimulations. These areas were then plotted against the number of preceding stimuli, as shown in Figure 2C. The relationship between stimulus frequency and rate-dependent block appeared generally to be linear. The slope of a linear regression line thus indicates the extent to which the area under the curve is reduced with further stimuli, and is an alternative way to quantify the extent of rate-dependent block. (A further potential advantage of the linear regression is that the 10, 15, 20 Hz readings then contribute to the estimate of the change at 30 Hz, which should minimize the influence of an occasional aberrant reading in the estimates of 30 Hz rate-dependent block).

**Figure 3.**
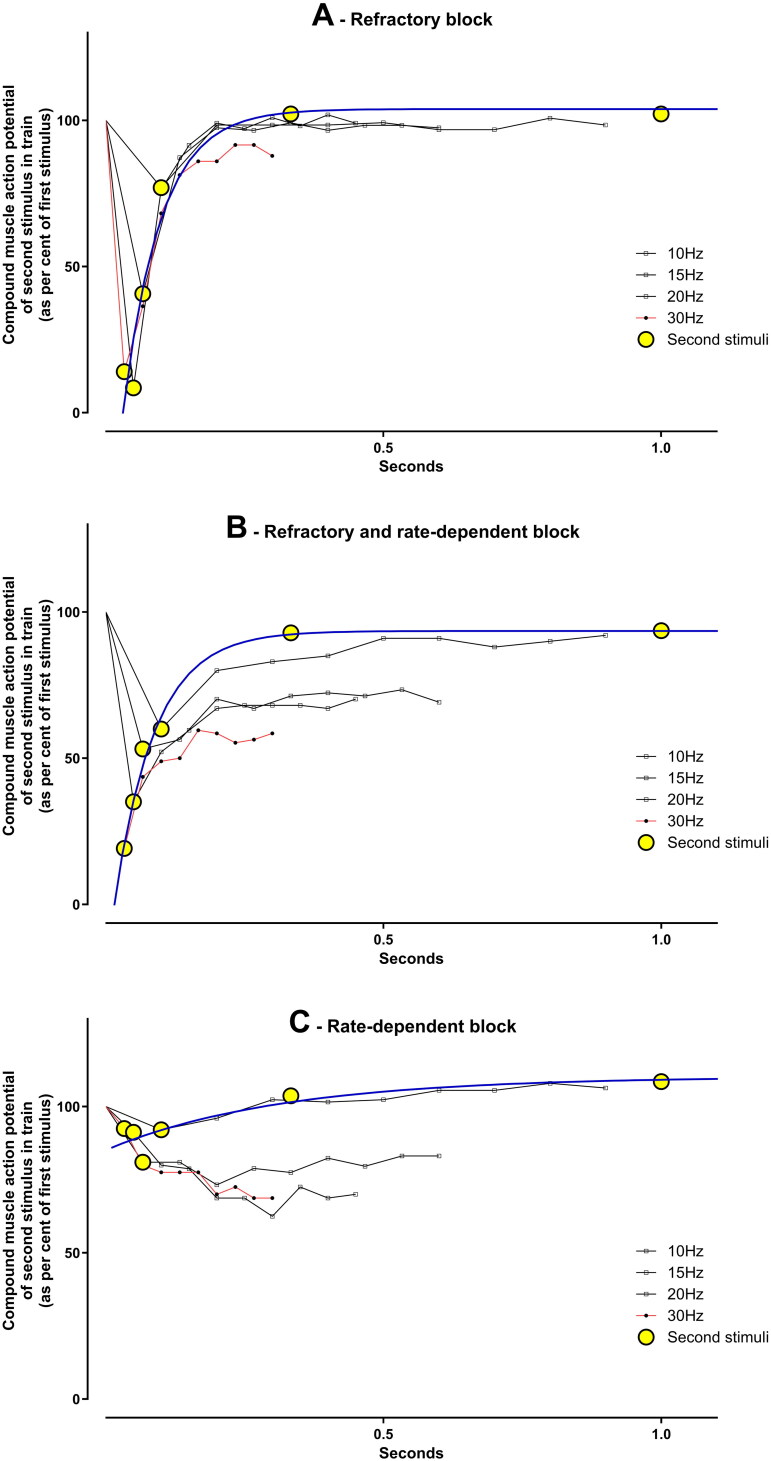
Sequential repetitive nerve stimulation changes in one patient with the intermediate syndrome demonstrating that the refractory and rate-dependent blocks can occur together (B) or in isolation (A,C). This patient with intermediate syndrome developed progressively increasing severe weakness after ingesting chlorpyrifos. A, B and C were taken at approximately 45, 62 and 92 h post-ingestion, respectively.

This allows a number of possible ways to more rigorously quantify the absolute and relative severity of the two blocks. The duration and severity of the refractory block can be quantified by the decrease in the cross-hatched area above the curve of the compound muscle action potential amplitude of the second stimulus at 3 to 30 Hz. This can be very simply done by the trapezoidal rule or use of the second stimulus_0.033 s_ and the k of recovery calculated from the non-linear regression of the one phase association equation [second stimulus_t_ = second stimulus_0.033 s_ + (100% − second stimulus_0.033 s_) * (1−e^(-kt)^)]. These give very similar results and we have therefore used the trapezoidal rule in the data in the Tables and Figures. The extent of rate-dependent exacerbation of block (increase in the area between the curves with increasing frequency of stimuli, i.e., the red-diagonal striped area in Figure 2B) could be quantified as the actual or estimated further reduction in area under the curve at 30 Hz repetitive nerve stimulation (Figure 2B) or the slope of the decline in area under the curve (Figure 2C). These two blocks could occur in the same patient at the same time or each largely in isolation. Figure 3 demonstrates an extreme example of this in one patient with intermediate syndrome who demonstrated all three permutations between 2 and 4 days after poisoning.

To examine whether these two distinct pathophysiological abnormalities were detectable on repetitive nerve stimulation in both intermediate syndrome and the *forme fruste* intermediate syndrome, we first plotted the reduction in compound muscle action potential areas attributable to the refractory and rate-dependent blocks against time for these patients. We then examined if either block was better associated with more severe weakness (intermediate syndrome) and respiratory failure (intermediate syndrome requiring ventilation) by comparing the maximum block in the intermediate syndrome patients with that of the less severe *forme fruste* intermediate syndrome patients using the Mann Whitney test.

**Figure 4.**
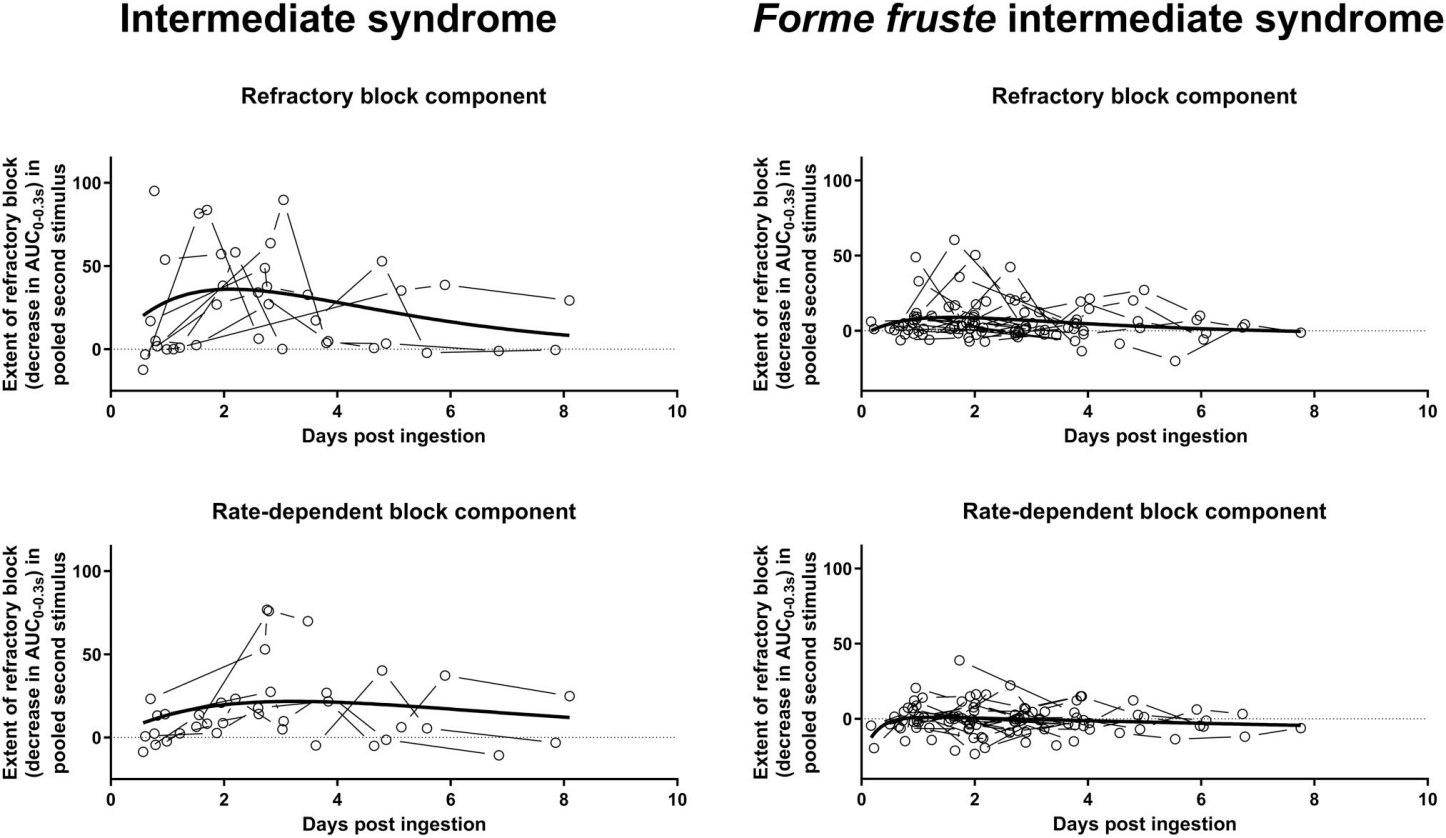
Progress of neuromuscular junction block as measured by repetitive nerve stimulation at 30 Hz changes over time in 10 individuals with intermediate syndrome and 24 individuals with *forme fruste* intermediate syndrome (127 repetitive nerve stimulation studies in total). All data are normalized to the first stimulus reading. A curve created with non-linear regression of a Bateman curve (Y = Span1*ka/(ka-k1)*[exp(-k1*x)-exp(-ka*x)]+Plateau) is shown to approximate the mean effect and time course without implying a specific mechanism.

All types of blocks were more commonly observed and more persistent and severe in those with intermediate syndrome (with or without respiratory failure) (Figure 4). However, the difference was most apparent for rate-dependent block. Only one reading from someone with the *forme fruste* intermediate syndrome had more than a 25% reduction in compound muscle action potential amplitude area attributable to rate-dependent block compared to nine readings from five of the patients with intermediate syndrome, and this included four of the five people with weakness requiring ventilation.

**Figure 5.**
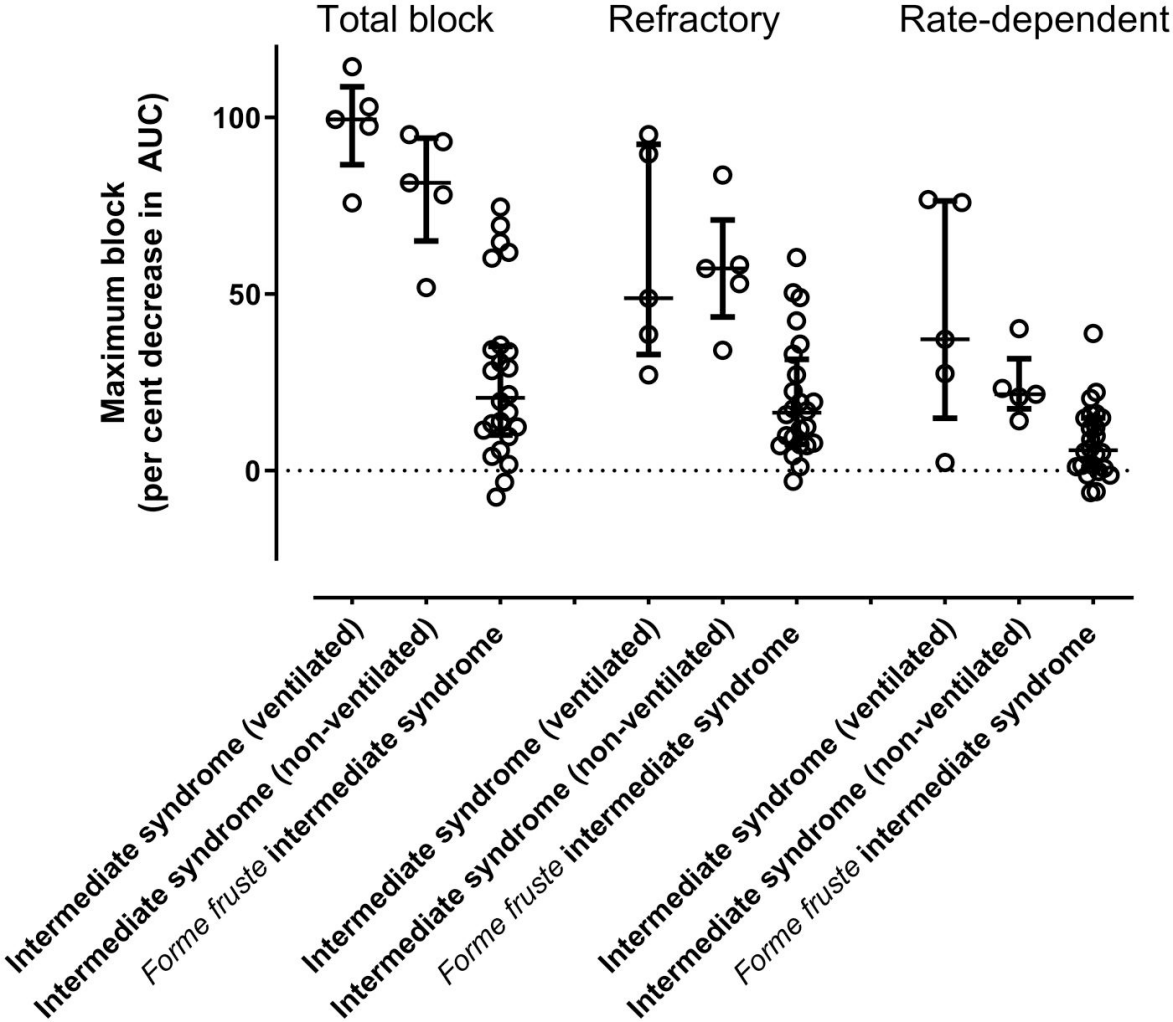
Maximum refractory, rate-dependent and combined block demonstrated on repetitive nerve stimulation in 10 patients with intermediate syndrome and 24 patients with *forme fruste* intermediate syndrome. All differences between pooled patients with intermediate syndrome and patients with *forme fruste* intermediate syndrome were highly statistically significant (*P* ≤ 0.003, Mann-Whitney test). Bars represent Median and IQR and standard error in the mean.

While numbers were limited, statistical analysis confirmed all types of block were significantly more severe in intermediate syndrome patients than those with just *forme fruste* intermediate syndrome (Figure 5 and Table 2).

**Table 2.** Comparison of quantification of block by different methods for the ten patients with intermediate syndrome and 24 with *forme fruste* intermediate syndrome (at the time of worst block).

Electrophysiological readings at time of maximum block	Intermediate syndrome (*n* = 10)	*Forme fruste* intermediate syndrome (*n* = 24)	*P*-value
Refractory (pooled second stimulus) block *	55% (34 to 84%)	16% (7 to 33%)	0.0001
Minimum second stimulus *	18% (8 to 25%)	45% (32 to 74)	0.0005
Rate dependent block (by subtraction)	20% (11 to 34%)	4% (0 to 14%)	0.003
30Hz block *	89% (73 to 94%)	21% (4 to 55%)	<0.0001
20Hz block *	75% (66 to 96%)	19% (7 to 29%)	<0.0001
15Hz block *	63% (51 to 93%)	16% (7 to 33%)	<0.0001
10 Hz block *	58% (34 to 86%)	16% (5 to 28%)	<0.0001
Rate-dependent block (using slope decline)	30% (13 to 46%)	7% (0 to 17%)	0.001
Total block	90% (78 to 98%)	23% (10 to 37%)	<0.0001

All data are expressed as medians (IQR).

* All expressed in units of per cent of first stimulus.

# Mann Whitney test.

## Discussion

There appear to be (at least) two distinct pathophysiological abnormalities detectable on repetitive nerve stimulation in organophosphorus poisoning. These may both be present when the classic “decrement-increment” phenomena are observed, but these phenomena can have a similar appearance with the more common refractory block in isolation. These deficits are quantifiable and appear to correlate well with the severity of respiratory muscle dysfunction. The rate-dependent loss of compound muscle action potential amplitude is associated with more severe weakness and a much higher risk of respiratory failure.

We had previously observed that the degree of muscle weakness correlated well with the severity of electrophysiological features during the progression of intermediate syndrome and that when patients developed respiratory muscle weakness, there was sometimes a transition of the electrophysiological features from decrement-increment to more severe or progressive decrements at high frequencies. This study demonstrates that this transition is a reflection of a severe rate-dependent block. However, less severe degrees of this block could occur in the presence of an apparent decrement-increment pattern (e.g., Figure 2). Yet this block generally occurred only in the group who at some stage fulfilled diagnostic criteria for classical intermediate syndrome, suggesting that earlier detection of this block might be clinically useful in identifying patients at risk of late respiratory failure. Further, while previously we have indicated that clinical intermediate syndrome appears to be a spectrum disorder, these data provide some evidence that there might be a clear distinction pathophysiologically between those with mild to moderate weakness and those with severe intermediate syndrome. The two pathophysiological abnormalities being demonstrated can only be speculated on but are most likely due to persistent inhibition of acetylcholinesterase activity. Repetitive nerve stimulation findings in the rare congenital acetylcholinesterase deficiency are similar and have been postulated to be explained by both pre-synaptic (e.g., decreased choline substrate for acetylcholine production, negative feedback, damage to nerve terminal) and post-synaptic (e.g., end-plate damage or persistent partial depolarization, receptor desensitization) responses to persistently high concentrations of acetylcholine [16]. In animal studies, acetylcholinesterase inhibition at the neuromuscular junction may lead to both depolarization and desensitization block [13]. Desensitization was observed with the decrement pattern of repetitive nerve stimulation. Persistent accumulation of acetylcholine (or acetylcholine agonists such as succinylcholine [17]) leads to a reduction of the number of functioning nicotinic receptors at the post-synaptic junction. This may be due to downregulation or conformational change of the receptor in response to acetylcholine, but there may also be direct effects of high concentrations of organophosphorus insecticide at the neuromuscular junction [18]. Some organophosphorus insecticides have been demonstrated to have a much higher incidence of intermediate syndrome [19,20].

However, a decrement pattern may also represent the pre-synaptic effects of acetylcholine or organophosphorus insecticide on rapid vesicular recycling or limiting acetylcholine substrate influx or production. No abnormal patterns of repetitive nerve stimulation are specific to either pre- or post-synaptic pathology.

Depolarization block may be due to antidromic backfiring arising from stimulation of pre-synaptic nicotinic receptors. Pre-synaptic depolarization block may be antagonized with magnesium and low doses of pancuronium, both of which have been shown to apparently reverse the decrement-increment pattern in organophosphorus poisoning [21]. These treatments may directly impair neuromuscular junction function and there is no evidence that reversal of the decrement-increment pattern or these treatments leads to short- or long-term clinical benefits. Further, in this small study, electrophysiological improvement was not accompanied by an improvement in muscle strength. This raises the question as to whether the decrement-increment phenomena is an important part of the pathophysiology of intermediate syndrome or simply an epiphenomena that may in fact mask the true pathophysiology(s). However, a more recent clinical study showed that neuromuscular junction electrophysiology seems to be important in clinical settings; single-fibre EMG findings correlated with both development of intermediate syndrome-related respiratory failure and the time of subsequent extubation [10]. Serial studies which separate out the electrophysiological components in future studies, along with the use of various agents that modify neuromuscular junction function, may greatly help to further understand the pathophysiology as it develops and relate it more closely to the clinical signs.

Animal models of intermediate syndrome would be helpful to fully explore each of these potential sources of variability. Surprisingly, while there are many studies that demonstrate immediate neurophysiological changes with anticholinesterases such as neostigmine [13], there are no published animal models that have demonstrated a prolonged severe neuromuscular junction dysfunction with the onset 1 to 4 days after poisoning. The delayed onset of synaptic pathology around 24 hours post organophosphorus exposure has been clearly demonstrated, but not the nature of the corresponding findings on electrophysiology [22]. Thus, it is unclear whether these animal studies are exploring the pathophysiology of intermediate syndrome or the less persistent and generally milder weakness seen with early cholinergic effects. In any case, our findings provide another means by which electrophysiological findings can be used to explore pathophysiology in humans. This technique may also be useful in testing the validity of any proposed animal model of intermediate syndrome, which ideally should replicate the time course and quality of neuromuscular junction effects seen in humans.

The quantification method resulted in some negative readings for block due to rate-dependent facilitation ("pseudo-facilitation"), a well-described finding in normal studies. It also led to estimates of total block slightly greater than 100% when very high degree block was observed at lower frequencies than 30 Hz. Thus, this scale is not simply a numerical percentage but is better regarded as an ordinal scale of severity. The area calculations were only performed out to 0.3 s. However, it is clear in a few very severe refractory blocks that there was substantial loss of compound muscle action potential amplitude and continuing block beyond this time (in severe cases, the second stimulus at 1 Hz (1 s) was reduced substantially. Thus, the scale may under-rate the change in severity at the high end of the scale (i.e., the difference between "70%" and "100%" block may be much greater than that between "10%" and "40%"). Moreover, the units of rate-dependent and refractory block should not be assumed to be clinically or electrophysiologically equivalent. A "25%" rate-dependent block could cause much greater impairment of muscle contraction than a "25%" refractory block. As further data correlating changes in muscle power with electrophysiology are obtained, it may be necessary to consider if modification of the units of either scale might improve the clinical relevance of the repetitive nerve stimulation results.

As for the clinical implications, our findings suggest that total block and the rate-dependent block at 30 Hz stimulation discriminated well between those who develop full-blown intermediate syndrome and milder forms of weakness (Table 2). The 30 Hz frequency is physiologically relevant to normal muscle activity [23]. This is in contrast to much slower frequencies, such as those used in "train of four" [24], which would have been normal in most of our patients’ readings. It is possible that other methods designed to focus on the decrement response, such as the ninth stimulus/first stimulus ratio at 50 Hz repetitive nerve stimulation [14], would similarly correlate well with clinical intermediate syndrome. However, this has not been studied in conscious patients with organophosphorus poisoning. It is not clear how well it would be performed or be tolerated as a screening test prior to ventilation.

It should be simple to explore the potential of administering a cut-down version of our repetitive nerve stimulation protocol to predict intermediate syndrome/delayed respiratory failure: that is to test a patient with a single train of 30 Hz and calculate the total block (a direct output of the repetitive nerve stimulation protocol in modern EMG setups). This could be enhanced with appropriate programming by a series of paired stimuli at lower frequencies (1, 3, 10, 15, 20 Hz) to estimate the refractory block – the total protocol could involve 20 electrical stimuli rather than the 60 used in our patients – and the later paired stimuli would only need to be done in those with an abnormal 30 Hz reading. This protocol will be more tolerable for patients and feasible for clinical neurophysiologists to use in bedside testing of these patients, allowing prompt reporting of the results.

## Limitations

An important limitation is that we show findings from only 34 people and there were limited repetitive nerve stimulation studies performed on each individual. These were sufficient to demonstrate proof-of-concept, but further studies with more patients are needed to better define the correlates, clinical relevance and possible diagnostic/prognostic roles for the use of this technique to separately quantify the two neuromuscular junction blocks. The red blood cell acetylcholinesterase activity and the concentrations of chlorpyrifos and pralidoxime were measured in many of these patients, but blood was not always taken at the precise time of nerve conduction studies. Thus, while we have previously shown a correlation between the severity of neuromuscular junction dysfunction and the cumulative exposure or effect over time, in this study, we were not able to closely relate changes observed on repetitive nerve stimulation to the red blood cell acetylcholinesterase activity at the same moment.

This study included only one patient known to have ingested an organophosphorus insecticide other than chlorpyrifos. Intriguingly, this patient who ingested dimethoate had the most severe block of any patient in this cohort. Further studies are required to see if these two blocks are found with other organophosphorus insecticides.

Oximes might, in theory, prevent the development of intermediate syndrome, but this was beyond the scope of this study as nearly all the patients received the same intermittent low-dose pralidoxime for approximately 48 h. Ideally, further prospective studies will use this method to better quantify the response of the neuromuscular junction over time and in response to various treatments, as current data on such responses is limited and inconsistent [11,21]. It might also be used to compare different organophosphorus insecticides with different expected responsiveness to oximes in terms of acetylcholinesterase reactivation. This may guide the dosing of oximes to achieve a better benefit/risk ratio, as current recommended doses do not lead to the expected clinical benefits [25].

## Conclusions

Electrophysiological quantification of both refractory and rate-dependent block may provide valuable new tools to facilitate clinical and research assessment of the development of intermediate syndrome. These need to be validated as useful for patient management in future prospective studies, but our preliminary analysis strongly suggests that separating out the rate-dependent block may give a much clearer indication of those who will develop the most severe block and require ventilation. We hope these findings will also stimulate further animal and clinical studies to determine the pathogenesis of intermediate syndrome which may in turn help to improve the management of organophosphorus poisoning.
